# Supplementary figures and images for: The Results After One Year of an Experimental Protocol Aimed at Reducing Paratuberculosis in an Intensive Dairy Herd
Source: Animals (Basel). 2025 Sep 15;15(18):2695. doi: 10.3390/ani15182695 (PMC12466710; doi:10.3390/ani15182695)

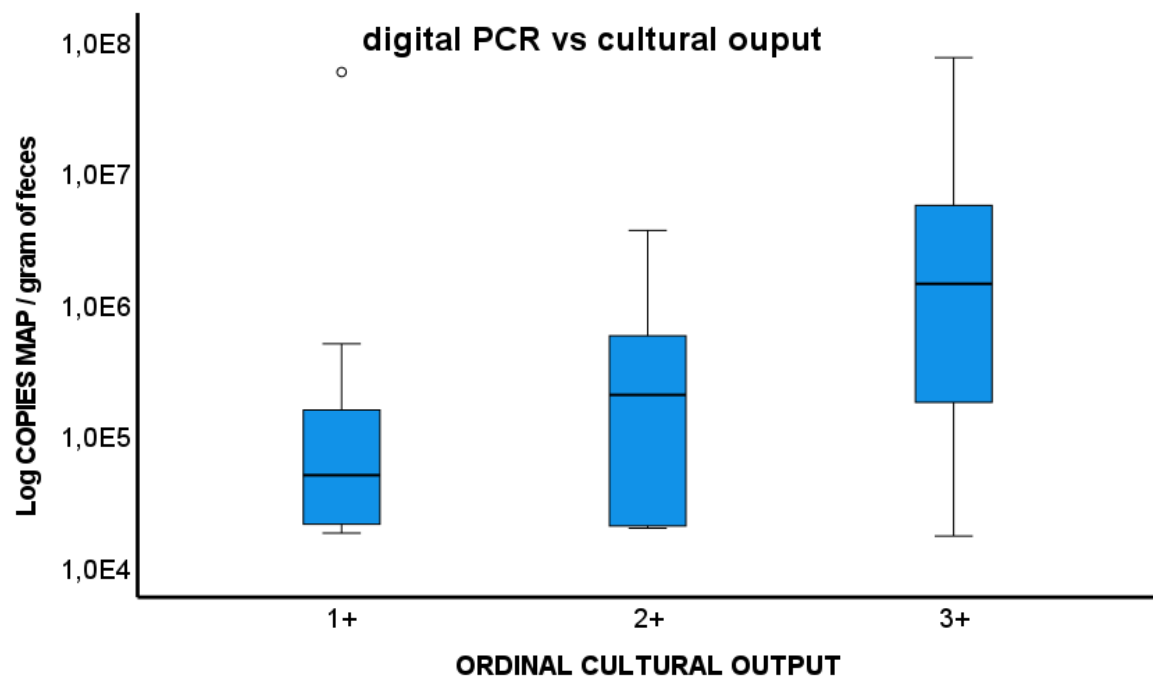

Supplement: Supplementary file 1 [file animals-15-02695-s001.zip › Filippi et al.,Figure S1.pdf]
